# Honeybees Exposure to Natural Feed Additives: How Is the Gut Microbiota Affected?

**DOI:** 10.3390/microorganisms9051009

**Published:** 2021-05-07

**Authors:** Daniele Alberoni, Loredana Baffoni, Chiara Braglia, Francesca Gaggìa, Diana Di Gioia

**Affiliations:** Department of Agricultural and Food Sciences (DISTAL), University of Bologna, Viale Fanin 44, 40127 Bologna, Italy; daniele.alberoni@unibo.it (D.A.); chiara.braglia4@unibo.it (C.B.); francesca.gaggia@unibo.it (F.G.); diana.digioia@unibo.it (D.D.G.)

**Keywords:** honeybee, gut microbiota, bacteria, probiotics, thymol, oceanic algae, *Bartonella*, *Bombilactobacillus*, *Snodgrassella*

## Abstract

The role of a balanced gut microbiota to maintain health and prevent diseases is largely established in humans and livestock. Conversely, in honeybees, studies on gut microbiota perturbations by external factors have started only recently. Natural methods alternative to chemical products to preserve honeybee health have been proposed, but their effect on the gut microbiota has not been examined in detail. This study aims to investigate the effect of the administration of a bacterial mixture of bifidobacteria and Lactobacillaceae and a commercial product HiveAlive^TM^ on honeybee gut microbiota. The study was developed in 18 hives of about 2500 bees, with six replicates for each experimental condition for a total of three experimental groups. The absolute abundance of main microbial taxa was studied using qPCR and NGS. The results showed that the majority of the administered strains were detected in the gut. On the whole, great perturbations upon the administration of the bacterial mixture and the plant-based commercial product were not observed in the gut microbiota. Significant variations with respect to the untreated control were only observed for *Snodgrassella* sp. for the bacterial mixture, *Bartonella* sp. in HiveAlive^TM^ and *Bombilactobacillus* sp. for both. Therefore, the studied approaches are respectful of the honeybee microbiota composition, conceivably without compromising the bee nutritional, social and ecological functions.

## 1. Introduction

The importance of bees for the maintenance of the planet’s biodiversity and the functionality of the agroecosystem is globally recognized [1,2,3]. Honeybees (*Apis mellifera*), in particular, are known to provide valuable ecosystem services, fruit and crop pollination and are also reared for honey and other hive products. Honeybees are often moved over long distances for both pollination needs [4,5] and honey harvest (transhumance), exposing them to possible stressors, and contributing to the spread of diseases. Therefore, honeybees can no longer survive without human constant inputs, especially in disease control and emergency nutrition [6,7]. Pathogens and parasites play the greatest role in bee diseases, although they often act in synergy with a multitude of abiotic stressors. The most serious honeybee diseases are caused by *Paenibacillus larvae* [8], *Melissococcus plutonius* [9], *Nosema apis* and *Nosema ceranae* [10,11] and *Varroa destructor* and are vectored by the mite honey bee-associated viruses. Colony decline is accelerated by several abiotic stressors, such as atmospheric or soil pollutants, plant protection products (e.g., pesticides) and climate change altering nectar availability or bee biological life-cycle [12,13]. For these reasons, in the last century, many beekeepers have started to rely on antibiotics against some pathogens. Unfortunately, honeybee diseases have not ceased their virulence and the observed high colony mortality rate has not been reduced in some regions, like Europe. Moreover, the latest restrictions in Europe and the USA on the use of antibiotics [14,15,16,17] have encouraged the search for alternative approaches to antibiotics by researchers and private companies. Consequently, new strategies based on the use of plant extracts such as thymol [18,19], eucalyptol [20,21], or bacteria with probiotic potential and the use of prebiotics ingredients [22,23] have been proposed as possible mitigation strategies, and some products are already on the market. Recent studies have shown the importance of the honeybee gut microbiota for immune system stimulation, resistance to pathogen colonization, and for the digestion of pollen and sugars [24]. So far it is known that the bacterial gut community of the honey bee includes by five to nine taxa, each corresponding to a species or a cluster of closely related species. Usually, two clusters of Lactobacillaceae species collectively referred as the “*Lactobacillus* Firm-4” (*L. mellis* and *L. mellifer*) and “*Lactobacillus* Firm-5”(*L. apis*, *L. helsingborgensis*, *L. kullabergensis*, *L. kimbladii* and *L. melliventris*) are the most abundant, followed by *Bifidobacterium* spp., (*B. asteroides*, *B. coryneforme* and *B. indicum*); “Gamma-1” (*Gilliamella apicola*) and “Gamma-2” (*Frischella perrara*); “Alpha-1” (*Bartonella apis*), ‘Alpha-2.1’ (Acetobacteraceae) and Alpha-2.2’ (*Parasaccharibacter apium* or *Bombella apis*) [25,26]. Despite its simple core composition, the gut microbiota shows a great genetic variability. As an example, the species *Snodgrassella alvi* has a similarity of homologous protein-coding genes ranging from 80 to 90%, thus showing diverse gene pool among strains [27]. Gut microbiota can be affected by various factors such as exposure to chemical compounds (weed killers or antibiotics) [28,29,30], season and age, that may challenge honeybee well-being. The effect on the gut microbiota, and therefore on honeybee health, of commercial feed additives, even if organic and apparently safe, cannot be predicted. In this experimental research, we investigated the effect on the honeybee gut microbial community of two feed additives: a bacterial mixture (BM) of Lactobacillaceae and bifidobacteria already designed and preliminary tested in field as stimulant to increase productivity of honeybees [23], and a commercial product based on thymol and seaweeds extracts (HiveAlive^TM^). The present study, differently from the previous one [23], is designed with multiple experimental replicates and targets nourse honeybees at a precise age. The microbiota were studied in new generation of bees born after the end of the treatments and compared with the gut microbiota at the beginning of the experiment.

## 2. Materials and Methods

### 2.1. Experimental Design

**Figure 1:**
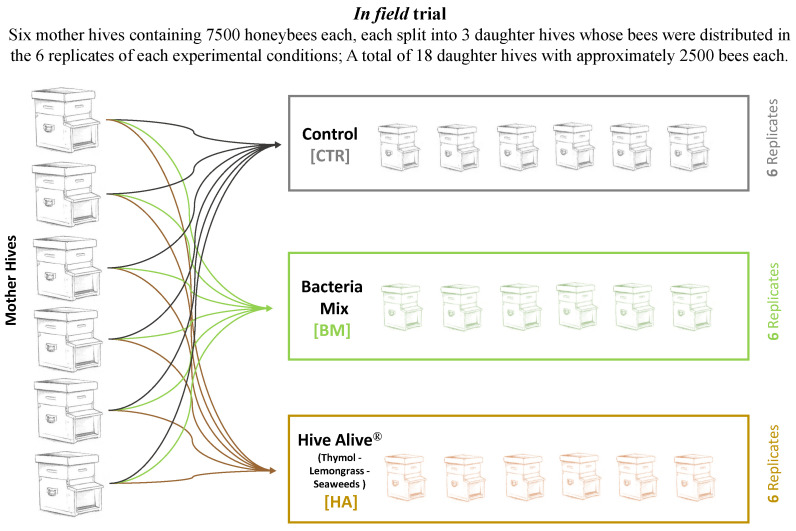
Experimental Design. Schematic representation of the field trial. The figure reports the scheme of the tests and the number of bees and beehives used in the trials.

The bacterial mixture and the plant-based product were tested in field in a trial performed between July and August 2017 with 6 replicates per experimental conditions. The trial was set up at the experimental apiary of the University of Bologna at San Lazzaro (Bologna, Italy). Honeybee experimental nucs for the trial were prepared by dividing 6 fully developed mother colonies Dadant-Carlini [31] into nucs of approximately 2500 honeybees each. The equal distribution of the 6 honeybees’ colony genetic profiles into the different experimental conditions ensured that each genetic profile was represented and homogeneity of the resulting hives. Moreover, care was addressed in distributing fresh brood in every nuc, in order to standardize the pupae emerging time among nucs. Finally, freshly mated queens were immediately provided upon nucs formation. The experiment resulted in a total of 6 nucs per thesis (experimental replicates, Figure 1). Each nuc was fed once a week with 500 mL of sucrose solution 1:1 (w:w). A total of 4 feedings were applied to compensate the lack of natural nectar. 18 honey bee colonies were divided into three experimental groups: (1) [HA], in which HiveAlive^TM^, a commercial product containing thymol, lemongrass and seaweeds (provided by Advance Science Ltd, Galway, Ireland), was administered mixed with sugar syrup (1:1 *w*/*v*); (2) [BM], in which a bacterial mixture was provided resuspended with sugar syrup (1:1 *w*/*v*), and (3) [CTR], the control, with no active ingredient or bacteria applied but with sugar syrup (1:1 *w*/*v*). During the trial, two foraging conditions were present: *Metcalfa pruinosa* honeydew at the beginning of August and *Medicago sativa* blooming all throughout the experimental time even if strongly limited by summer drought. All mother hives were treated at the end of June with a trickle application of a 4.2% solution of oxalic acid; the obtained nucs health status was periodically assessed (varroa infestation and virus symptomatology, adult honeybee population, brood size and honey reserves) and any relevant variation was annotated.

### 2.2. Treatments and Samplings

HiveAlive^TM^ was administered according to the manufacturer’s instructions, as summarized in Table 1. BM is a mixture of 6 strains, belonging to the *Bifidobacterium* genus and the Lactobacillaceae family, developed and preliminary tested on bees in previous studies [23,32]. In particular the strains used were *Bifidobacterium asteroides* C3 (DSM20431), *Bifidobacterium coryneforme* C155, *Bifidobacterium indicum* C449, *Apilactobacillus kunkeei* Dan39, *Lactiplantibacillus plantarum* Dan91, *Lactobacillus johnsonii* Dan92. BM was prepared freshly each day of administration at a concentration of 2.2 × 10^8^ bacterial cells/mL according to Alberoni et al. [23], also briefly summarized in Table 1. Honeybees and combs were sprayed once a week for three weeks with 30 mL of sugar syrup (1:1 w/w) containing the prepared treatment (HA or BM). In this way, all bees present in the hive received the treatment, whereas the remaining 3% to 10% forager bees received the treatment via trophallaxis. Finally, 50 emerging honeybees per replicate were marked on the thorax [33] with non-toxic coloured nail polish in the days immediately following the 3rd treatment (days 15–17). The sacrifice of marked honeybees was performed at day 24, when honeybees resulted at nurse stage (7–9 days post eclosure). According to the literature, in this phase bees possess a completely established gut microbiota [34]. Thirty honeybees per replicate were sampled and the gut content pooled at the beginning of the experiment (T0) and after 24 days (T1) (a total of 180 bees/experimental condition and time). The dissection was performed using tweezers by pulling the last tergites and stinger to extract the digestive system. The midgut, pylorus, ileum and rectum were used for further analysis, whereas the crop was dissect with a scalpel when necessary. The experimental trial was organized in 2 sampling times, T0 and T1, for 3 experimental conditions (CTR, BM and HA) each composed of 6 replicates. Each sample is indicated with the acronym: sampling time_experimental condition_number of replicate.

### 2.3. DNA Extraction and NGS Sequencing

Obtained gut content pools were homogenised with a pestle, mixed with 1400 μL lysis buffer (Zymo Research, Tustin, California, USA) and 60 μL proteinase K (AppliChem GmbH, Darmstadt, Germany) at a final concentration of 20 mg/mL. Samples were further broken down with glass beads shaking at 50 Hz, and followed by 1-h incubation at 55 °C. 450 μL of the resulting sludge was used for gut genomic DNA extraction with Quick-DNA Fecal and Soil Microbe Kit (Zymo Research, California, USA). A total of 36 samples were subjected to NGS analysis on Illumina MiSeq platform (BioFab s.r.l, Rome, Italy). The amplification of V3-V4 region of 16S rRNA gene, libraries preparation for Illumina MiSeq platform and sequencing were performed according to Alberoni et al. [35] as well as bioinformatic analyses, which relied on the most updated SILVA database release 132. The database was implemented inserting full length 16S rRNA sequences of administered bacteria. OTUs with less than 0.1% abundance were discarded. alpha–diversity was evaluated using Chao1, Observed OTU and PD whole tree metrics, whereas beta–diversity was evaluated using both weighted and unweighted UniFrac.

### 2.4. Quantification of Target Microbial Groups

Total bacteria, Lactobacillaceae and *Bifidobacterium* spp. were quantified with qPCR (StepOne™ Real-Time PCR System, Applied Biosystems) according to Baffoni et al. [36]. Data for Lactobacillaceae (*Apilactobacillus* spp., *Bombilactobacillus* spp., *Lactobacillus* spp. and *Lactiplantibacillus* spp.) and *Bifidobacterium* spp. were analysed to obtain the number of microorganism as Log CFU/single intestinal content [37,38]. For total bacteria data were expressed as Log 16S rRNA copies/intestine [39].

### 2.5. Data Adjustments and Classification of Microbial Genera

Rarefied biom tables obtained from NGS bioinformatic analysis were further adjusted—according to Raymann et al. [28], the absolute abundance of the most representative taxa was calculated using total bacteria qPCR output. Moreover, species belonging to the Lactobacillus genus have been recently re-classified [40], therefore, *Lactobacillus* spp. Firm-4 was manually re-classified to *Bombilactobacillus* spp. as well as the former *Lactobacillus kunkeei* and *Lactobacillus plantarum* to the new respective taxonomic classifications *Apilactobacillus kunkeei* and *Lactiplantibacillus plantarum*. The curated dataset was used for statistical and graphical analyses.

### 2.6. Statistical Analysis

Statistical analysis for NGS and qPCR data was performed with the R software [41] according to Alberoni et al. [35]. Normal and homoscedastic data were analysed with ANOVA, whereas a generalized linear model (glm) was used for non-normal, homoscedastic data (with normal distribution of residuals). Kruskal-Wallis test was used to analyse data with high deviation from normality (non-parametric data), coupled with Dunn-test. LDA Effect Size (LEfSe) was performed on Galaxy/HutLab online tool, to emphasize both statistical significance and biological relevance for the detected taxa. Finally, biological relevance was also tested with Cramér’s V [42] relying on packages rcompanion, vcd, psych, desctools and epitools. QIIME statistical elaboration was used for beta–diversity index, according to Alberoni et al. [35]. Post-hoc test among different groups was carried out and Bonferroni’s correction was applied. The post-hoc test considered pairwise comparisons within thesis, taking into consideration the impact of each treatment at the same time (experimental conditions uniformity at T0 or divergence at T1) and over time. Therefore, eleven comparisons were considered for this experimental research, also considering the control as a further treatment to monitor and evaluate the normal gut microbial community evolution resulting from the interaction of honeybees with the environment. Graphs were generated with ggplot2, ggpubr and Microsoft Excel. PCA analysis was performed using packages FactoMineR [43] and factoextra [44], taking into consideration 67 taxa at the species level, therefore also attributing importance to minor taxa (otherwise referred as Other_taxa along the paper). Confidence ellipses are shown in the graphs.

## 3. Results

### 3.1. General Observations on the Colony’S Status Pre and Post Treatment

The health status of the treated honeybee nucs was generally good over the whole trial and no visible sign of disease was recorded in all experimental replicates. The environmental conditions during the experiment did not allow a sufficient harvest of nectar by honeybees, therefore periodic feedings were necessary, in particular nucs BM_3, HA_2 and HA_3 resulted in low honey reserves at the end of the experiment.

### 3.2. qPCR Quantification of Total Bacteria, Bifidobacterium and Lactobacillaceae in the Bee Gut

**Figure 2:**
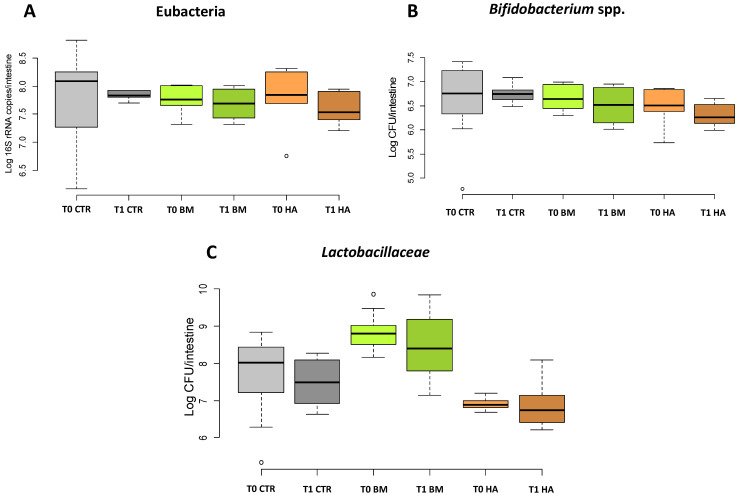
qPCR. quantification of (**A**) total bacteria (Eubacteria), (**B**) *Bifidobacterium* spp. and (**C**) Lactobacillaceae. Data are expressed in Log CFU/intestine for *Bifidobacterium* spp. and Lactobacillaceae; for Eubacteria data are expressed as Log 16S rRNA copies/intestine. [CTR] Control, [BM] Bacterial Mixture and [HA] HiveAlive^TM^.

A reduction of total bacteria was shown comparing T1 vs T0 of all treatments (CTR, BM and HA) (Figure 2A), although not significant. Also, *Bifidobacterium* spp. and Lactobacillaceae counts showed a similar trend, but no significant differences were recorded (Figure 2B,C).

### 3.3. Bee Gut Microbiota Analysis via NGS

**Figure 3:**
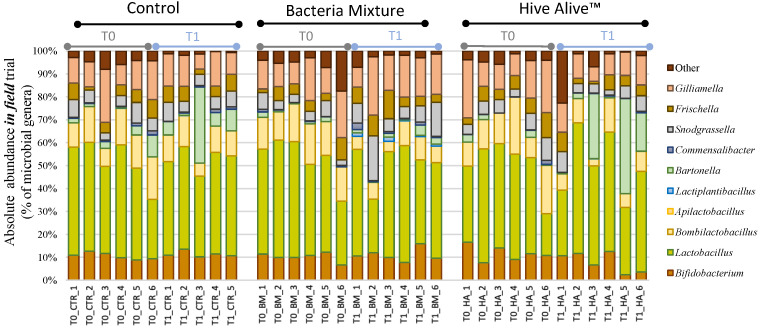
Absolute Abundance bar charts. Bar charts reporting the major cumulated microbial genera per samples and their absolute abundance expressed in percentage. Samples are reported with the sampling time T0 and T1, followed by the experimental conditions [CTR] Control, [BM] Bacterial Mixture and [HA] HiveAlive^TM^, and finally the replicate number (1–6).

About 10.3 million raw reads were obtained from the sequencing of 36 samples. Seven million reads passed the quality control and the Chimera check obtaining an average of 95,986 joint reads per sample and, for statistical analysis, samples were rarefied at 46,109 reads, a value obtained excluding the replicate T1_CTR_6 due to a particularly low coverage. The taxonomic assignment of the 32 samples produced 17,194 OTUs at 97% similarity and it was based on SILVA 132 database. The elaboration of NGS data is reported at phyla, family, genus levels in Table 2 and graphically at genus level in Figure 3.

At the species level, *Bartonella apis*, *Snodgrassella alvi*, *Gilliamella apicola*, *Commensalibacter intestini* and *Frischella perrara* confirmed the same absolute abundance and the same statistical results obtained for the corresponding genus (due to the presence of one species per each genus); therefore, the results for each treatment, in the next paragraphs, are not repeated for these species. Statistical analysis of alpha-diversity indices (Chao1, Observed OTU and PD whole tree) showed no significant variations, even if an increasing trend could be observed in BM group over time for all indices. A significant variation was obtained only in the weighted UniFrac analysis comparing the variability with time in CTR to the variability obtained in HA (Table 3).

No significant changes in the absolute abundances of the native bacterial taxa were detected in the control bees. All relevant changes for any taxonomic level are reported and summarized in Table 4.

**Figure 4:**
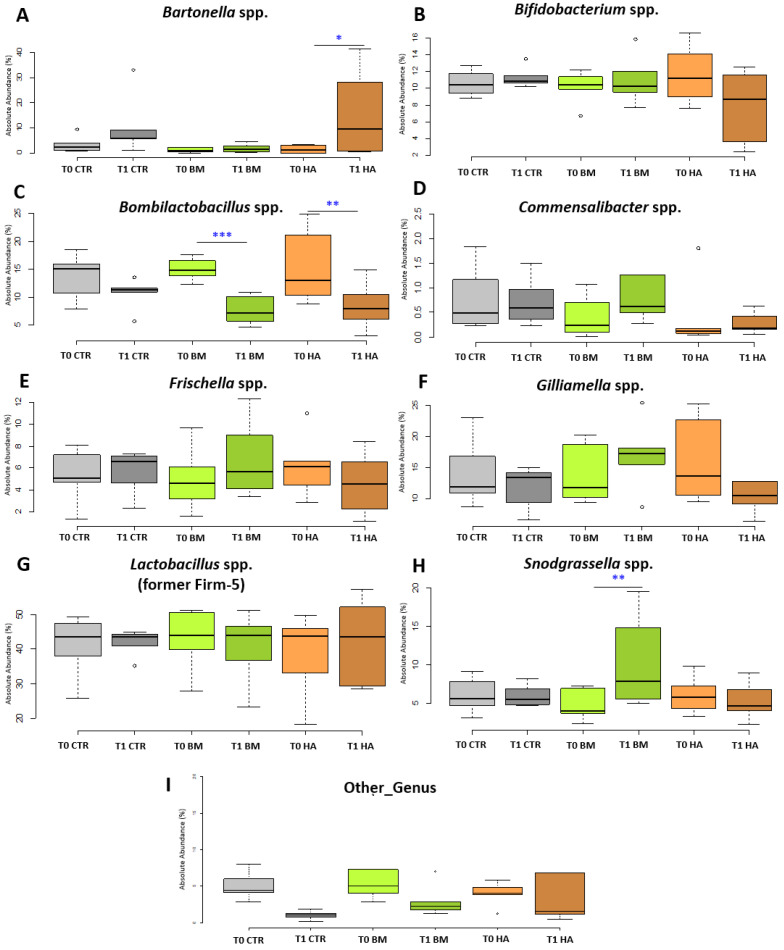
NGS Absolute Abundance box plots. Box plots reporting the major microbial genera expressed for their absolute abundance in percentage, and in relation to experimental conditions (significant pairwise comparisons * *p* < 0.1; ** *p* < 0.05; *** *p* < 0.01). Microbial taxa described: (**A**) *Bartonella* spp.; (**B**) *Bifidobacterium* spp.; (**C**) *Bombilactobacillus* spp.; (**D**) *Commensalibacter* spp.; (**E**) *Frischella* spp.; (**F**) *Gilliamella* spp.; (**G**) *Lactobacillus* spp.; (**H**) *Snodgrassella* spp. and (**I**) Other_genus. Experimental conditions [CTR] Control, [BM] Bacterial Mixture and [HA] HiveAlive^TM^.

All experimental conditions at T0 did not show any significant difference when compared among each other at any taxonomic level. At the family level, a significant increase was detected for Neisseriaceae (4.76% to 10.3%, *p* < 0.05) comparing BM_T1 vs BM_T0. Also, the comparison of HA_T1 vs HA_T0 showed a significant increase in Bartonellaceae (1.50% to 15.11%, *p* < 0.1). At genus level, *Bartonella* spp. significantly increased in HA_T1 vs HA_T0 from 1.53% to 15.08% (*p* < 0.1) but also in the CTR_T1 vs CTR_T0 even if not significantly Figure 4A. *Snodgrassella* spp. significantly increased in BM_T1 vs BM_T0 (*p* < 0.05) from 4.73% to 10.13%, reflecting Neisseriaceae family variation (Figure 4H) but also comparing BM_T1 vs HA_T1 (*p* < 0.1). On the other hand, *Bombilactobacillus* spp. showed a significant decrease for BM_T1 vs BM_T0 (from 15.00% to 7.61%, *p* < 0.01) and HA_T1 vs. HA_T0 (from 15.20% to 8.39%, *p* < 0.05—Figure 4C), a trend confirmed by the significant reduction of *Bombilactobacillus mellis* (*p* < 0.01) at species level in both BM and HA experimental conditions. *Lactiplantibacillus* spp. was significantly higher (*p* < 0.01) in BM_T1 group compared to all other experimental groups at T0 and T1. Even if not significant, it is worthy to report that *Gilliamella* sp. decreased from 15.92% to 10.33% in HA_T1 vs HA_T0 Figure 4F. All the experimental conditions at T1 did not show any significant variations when compared among each other at any taxonomic level with the only exception of *Lactiplantibacillus* spp.. In the same way comparisons of CTR_T0 vs BM_T1 or CTR_T0 vs HA_T1 did not show significant variations, when the p value is corrected with Bonferroni. According to the GLM and Kruskal-Wallis, the LEfSe analysis did not evidence any relevant variation among every experimental conditions at T0. On the other hand, comparing BM_T1 vs BM_T0, two taxa were over represented (*Snodgrassella* spp. and *Lactiplantibacillus* spp.) and two groups were under represented ( *Bombilactobacillus* spp. and Other_taxa), but only the two Lactobacillaceae taxa were significant (*p* < 0.05). *Snodgrassella* spp. was not significant when the Bonferroni correction was applied to LEfSE. The comparison HA_T1 vs HA_T0 did not show any relevant variation for any genus and species. Also the other considered comparisons did not show significant variations at genus and species level, with exception of CTR_T1 vs CTR_T0 that had a significant reduction of Other_taxa (*p* < 0.1) and BM_T1 vs CTR_T1 that presented a relevant increase of *Lactiplantibacillus* spp. (*p* < 0.1). LEfse Significant values are not reported in Figure 4. Finally, the Cramer’s V test showed a low biological relevance in pairwise comparisons of CTR_T1 vs CTR_T0 (Cramer’s V = 0.19), slightly moderate relevance for BM_T1 vs BM_T0 (Cramer V = 0.20 ) and moderate relevance in HA_T1 vs HA_T0 (Cramer’s V = 0.28).

**Figure 5:**
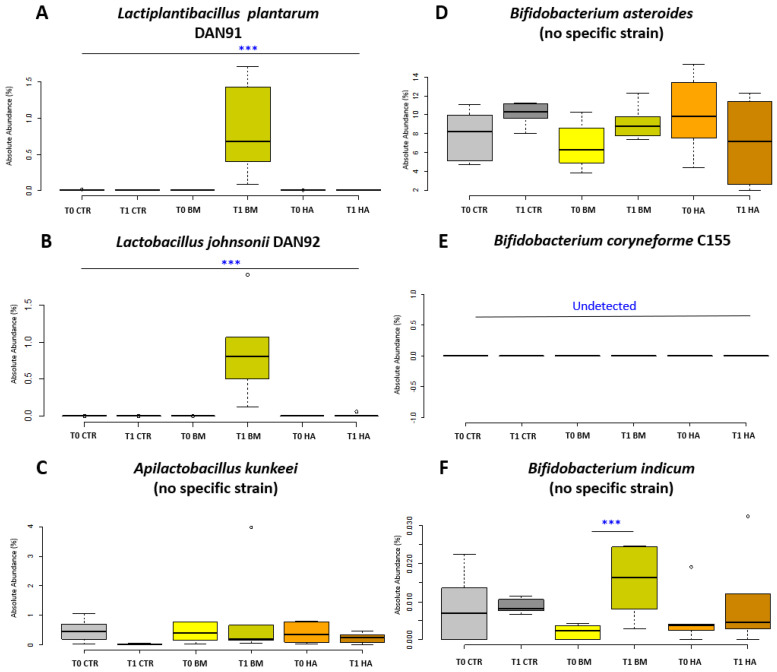
Absolute abundance of Bacteria Mixture in treated nucs. Box plots reporting the supplied microbial species composing the bacterial mixture, expressed for their absolute abundance in percentage and in relation to experimental conditions (significant pairwise comparison *** *p* < 0.01). (**A**) *Lactiplantibacillus plantarum* Dan91, (**B**) *Lactobacillus johnosonii* Dan92, (**C**) *Apilactobacillus kunkeei* Dan39, (**D**) *Bifidobacterium asteroides* C3, (**E**) *Bifidobacterium coryneforme* C155, (**F**) *Bifidobacterium indicum* C499. Experimental conditions [CTR] Control, [BM] Bacterial Mixture and [HA] HiveAlive^TM^.

Analysis at the species level of the BM experimental condition required a particular care to identify the bacterial strains supplied. Therefore, the SILVA database was implemented with specific full length 16S rRNA gene sequences deriving from a whole genome sequencing of all the strains of the BM. The identification of specific supplemented BM strains in the gut microbiome was partially successful—not all the supplemented strains could be discriminated as deriving from the BM due to an overlap between administered strains and indigenous strains of the honeybee microbiota. This was noted for *A. kunkeei*, *B. asteroides* and *B. indicum* that failed strain identification (illustrated in Figure 5 as “no specific strain”). Of the supplemented BM strains, *L. plantarum* increased significantly (*p* < 0.01; Figure 5A) as well as *Lactobacillus johnsonii* Dan92 (*p* < 0.01; Figure 5B) and *Bifidobacterium indicum* (*p* < 0.05; Figure 5F) when comparing BM_T1 vs. BM_T0. On the other hand, *Apilactobacillus kunkeei* Dan39 and *Bifidobacterium asteroides* C3 did not significantly change (Figure 5C,D). *Bifidobacterium coryneforme* C155 resulted undetectable at strain level discrimination in all experimental conditions. Other genera group showed a strong decrease in CTR_T0 vs. CTR_T1 and in BM_T0 *vs* BM_T1 even if data are not significant. In contrast, the same group increased in the comparison HA_T0 vs. HA_T1. No other significant variations were detected in microbial composition of honeybees treated with HiveAlive^TM^ over time at both genus and species level, except the significant reduction of *B. mellis* (*p* < 0.01) from 13.99% to 7.59%.

**Figure 6:**
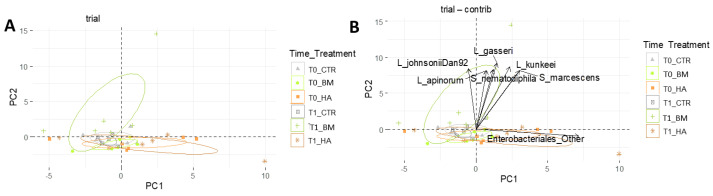
PCA analysis. (**A**) PCA without driver species relevance. (**B**) The graph (contrib) includes the variables with cos2 > 0.65 expressed in percentage.

PCA analysis at species level displayed a pattern of similarity among experimental conditions and sampling times. PC1 and PC2 together explained 25% of the variability and groups resulted not well separated and mostly dispersed along PC1 (Figure 6A). Therefore, the experimental conditions are not statistically distant and the core microbiota little perturbed after the treatments. “PCA contrib”, which represents the contribution expressed in percentage of the variables to the principal components [45], evidenced strain *L. johnsonii* Dan92, environmental lactobacilli and *Serratia* spp. as variables contributing to PC2 within BM_T1 group. These microbial taxa seem the major drivers of difference between BM_T0 and BM_T1 (Figure 6B). The graph also highlighted Enterobacteriales as a relevant variable to PC1, mostly associated with HA treatment. PCA analysis evidenced a non-significant shift of the honeybees microbiota, apart from the significant variation of few taxa, considering the overlap of confidence ellipses and the low percentage of principal components.

## 4. Discussion

The integrity of honeybee gut microbiota is important for the health and functionality of these pollinators. This work investigates the gut microbial community abundance and distribution in honeybees after the supplementation of selected Lactobacillaceae and *Bifidobacterium* strains and the commercial plant-based product HiveAlive^TM^ using qPCR and 16S rRNA next generation sequencing. The analysis is performed on a new generation of bees born after the feeding treatment. Lactobacilli and bifidobacteria are traditionally considered beneficial microbes in human and animals [46,47,48]. The selected strains (*L. johnsonii* Dan92, *A. kunkeei* Dan39 and *L. plantarum* Dan91, *B. asteroides* C3; *B. coryneforme* C155; *B. indicum* C449) have already been tested on honeybees with positive results against *N. ceranae* [32], and on healthy honeybees, showing an increase in hive productivity [23]. In the present study, a detailed analysis on the main gut microbial groups, also including single strain analysis when possible, has been performed. qPCR analyses did not highlight a great impact of the BM on the selected gut microbiota groups (Eubacteria, Lactobacillaceae and bifidobacteria). NGS absolute abundance confirmed qPCR results, with no *Lactobacillus* spp. (formerly Firm-5) variation during the treatment, in spite of the addition of *L. johnsonii*. No variations were detected also for *Bifidobacterium* spp. and this is partially coherent with results obtained by [49] that did not evidence an increase in the presence or load of the supplemented bacteria (*S. alvi*) when the analysis was performed at genus level. *Bombilactobacillus* spp. showed, on the contrary, a significant decrease, thus indicating that the administration of microbial strains to honeybees causes, when examined in details, some perturbations. The decrease resulted with high biological relevance also with LEfSe, confirming the importance of variation. *S. alvi*, residing in the ileum, increased after BM administration, probably thanks to the formation of a more favorable substrate availability derived from the sugar metabolism by the rectum population, for example, the presence of acetate [50,51]. Considering the three administered lactobacilli, *A. kunkeii* (formerly *L. kunkeii*) was present in all samples confirming its wide presence into the environment, especially in nectar and pollen. It accounts for a small proportion of the honeybee gut microbiota [52] as it is often detected in the crop rather than in the midgut and rectum [52,53]. *L. johnsonii* and *L. plantarum* were presumably acquired by trophallaxis and feces contact with the older honeybees, or by direct contact with the colony surfaces, making them well settled strains in the honeybee gut. It is interesting to point out how in PCA analysis only *L. johnsonii* Dan92 and *A. kunkeei* (presumably the administered strain Dan39) contributed to PC2 among all the supplemented strains. Other Lactobacillaceae, autochthonous for honeybees (e.g., *L. apinorum*) or of environmental origin (e.g., *L. gasseri*) and *Serratia* spp. were stimulated by the administration of BM strains. Considering that *S. marcescens* has been recently considered as an opportunistic pathogen of adult honey bees [54], the increase of members of the *Serratia* genus requires further investigations in relation to their pathogenic traits and to associate this increase to the BM administration.

Within bifidobacteria, *B. asteroides* is the most efficient colonizer of the bee gut being present at the same level at T0 in the CTR and BM samples. A ubiquitous presence is also observed for *B. indicum*, although its count is very low, increasing significantly in the gut at T1. However, considering that *B. coryneforme* detection failed in all samples and the close relatedness with *B. indicum* [55], it is probable that the analyzed sequences do not discriminate between the two species. On the other hand, the high detected amount of *B. asteroides* may indicate its capability of out-competing *B. indicum*, or that *B. indicum* is inhibited by other species present in the rectum. The administration of BM highlighted only minor changes, although not significant when Bonferroni correction was applied, in the microbial profiles comparing the treated hives against the CTR at both T0 and T1 and except for *L. plantarum*. Studies on humans and animals showed that beneficial bacteria may restore the composition of the gut microbiota in case of dysbiosis and support beneficial functions to gut microbial communities, resulting in amelioration or prevention of gut diseases and/or prevention of gut pathogens colonization. Beneficial microorganisms are not expected to significantly modify the intestinal microbial composition in healthy subjects, but to support the host to maintain the intestinal balance, and to support an immune-stimulation effect that can favor the host response to biotic and abiotic stresses [56,57]. The preservation of the host core microbiota, in this case of honeybees, is important to favor the host balance and stress response. The luck of wider variations in the gut microbiome should be considered as a positive result, because, on the contrary, a major shift in taxonomic groups or quantitative variations following bacterial supplementation may lead to a higher parasite susceptibility and may prevent the establishment of a robust microbiota [58]. Furthermore a suggested mechanism of action for beneficial bacteria is an increased tolerance to pathogens, for example, *N ceranae*, by improving honeybees immune system and tissue repair processes [59]. This is also supported by [60], who highlighted how endogenous bacteria could stimulate an immune response in honeybees.

Concerning the use of natural supplements of plant origin, many commercial products are available on the market that were shown to be active against *Nosema* spp. and valid alternatives to the use of the antibiotic Fumagillin [61,62]. However, natural extracts are not necessarily safe and may show toxicity when applied as feed additives. In this study HiveAlive^TM^ was chosen because it contains thymol, a well-known, broad-spectrum antimicrobial molecule used to counteract both *Nosema* spp. [63] and *Varroa destructor* [18,19], as well as seaweeds which are claimed as antimicrobial, immune system boosting and prebiotic agents [63]. Due to its strong antimicrobial activity, thymol contained in HiveAlive^TM^ was supposed to affect the gut microbial community [64]. Despite that, the administration of HA, as for BM, did not highlight significant variations in the microbial profiles comparing the treated hives against the CTR at both T0 and T1. However, comparing HA_T1 vs. HA_T0, our results show that HA was very well tolerated by the honeybee microbiota without causing any remarkable shift within the main core microbial taxa. The only two significant variations were the increase of *Bartonella* spp. and the decrease of *Bombilactobacillus* spp. absolute abundance. Focusing on the main active ingredients of HiveAlive^TM^ (thymol and seaweeds), the increase of *Bartonella* spp. can be ascribed to the capability of these bacteria of using plant secondary metabolites as a source of carbon and energy [65]. Interestingly, Castelli et al. [53,66] found a positive correlation between *B. apis* abundance and the feeding of *Eucaliptus grandis* pollen, containing a high amount of essential oils, among which is thymol [53,67]. *Bombilactobacillus* spp. is known to be affected by some plant protection products and other chemical compounds, such as antibiotics and weed killers [28,29,35,68], therefore it seems to be very sensitive to environmental perturbations. This may explain the decrease in the *Bombilactobacillus* absolute abundance when the plant-based commercial product is administered. In the same way, its decrease after the administration of the bacterial supplement may indicate difficulties of this microbial group in reacting or adapting to the presence of newly added strains. In BM treated colonies, the reduction of *Bombilactobacillus* may favour the increase of other taxa inhabiting the same ecological niche, such as *S. alvi*. A negative correlation between *S. alvi* and *Bombilactobacillus* sp. had already been observed by Kešnerová et al. [30].

## 5. Conclusions

In this work, the effects on the gut microbiota of bioproducts active against *N. ceranae* have been evaluated. To the best of our knowledge, an analysis of the honeybee gut microbiota following the exposure to plant- or microbial-based feed additives has never been carried out in detail, even though their beneficial impact has been documented [23]. The advancements with respect to previously published research [23] lie in the higher number of experimental replicates that makes the statistical analysis more robust, as well as in the precise determination of sampled honeybees age post BM administration. Moreover, the impact of a plant based product (HiveAlive^TM^) containing thymol, a natural compound very important in the beekeeping sector, has been studied for the first time in this work. The administration of HiveAlive^TM^ had a significant effect compared to CTR, and only on two microbial groups, one of which (*Bartonella* spp.) may have a positive effect for honeybee health as it is involved in plant secondary metabolites utilization. The administration of BM highlighted only minor changes in the microbiome profiles, comparing the treated hives to the respective controls. This is a positive result because beneficial microorganisms are not expected to significantly modify the intestinal microbial composition priming a dysbiosis. If the gut microbial composition of honeybees remains stable, the mechanism of action of BM may be explained by the stimulation of a immune response, that for BM treatments should be further studied and validated as indicator of honeybee colony health. On the whole, this research demonstrates that bio-based strategies, such as plant extracts or bacterial mixtures, and alternative to the use of chemotherapics, are respectful of honeybee gut microbiota, without determining any drastic change. Indeed, small detected changes can be positive for the bees, for instance, increasing their metabolic capabilities. Additional studies (involving metabolomics and shot-gun sequencing) aiming to determine the functionality of certain core microbial taxa are in progress to better elucidate the impact of specific gut microorganism variation.

## Figures and Tables

**Table 1:** Feed additives used in this work, their dosages applied in each treatment per hive in the presented trials, and recommended doses for full size colonies. * Dose recalculated according to the colony size of nucs, expressed as μL of active ingredient dissolved in 30 mL of sugar syrup. ** Total recommended dose for 3 administrations with weekly cadence; *** Prepared according to [23,32], 4 different administered with weekly cadence.

Experimental Theses			Dose per Treatment *	Reference Article
Thesis Acronym	Active Ingredient	Commercial Brand		
HA	Thymol + Seaweeds mixture	HiveAlive^TM^ Advance Science	300 L (2.4 mg of thymol)	Manufacturer instructions
BM	Bacteria mixture **	N.A.	2.2 × 10${}^{8}$ bacterial cells/mL ***	[23,32]
CTR	-	-	-	-

**Table 2:** Taxonomic assignment. Average values per time and treatment of microbial groups at phyla, family and genus level and related standard deviation.

	PHYLA							
	CTR_T0	CTR_T1		BM_T0	BM_T1		HA_T0	HA_T1
Actinobacteria	10.58 ± 1.49	11.37 ± 1.42		10.16 ± 2.01	10.96 ± 3.02		11.61 ± 3.23	7.92 ± 4.28
Firmicutes	55.12 ± 9.20	52.36 ± 6.71		57.90 ± 9.48	49.36 ± 10.85		54.28 ± 11.22	50.76 ± 14.62
Proteobacteria	29.30 ± 9.53	35.19 ± 7.89		24.95 ± 10.24	36.75 ± 9.74		30.11 ± 10.31	35.56 ± 17.26
Other_phyla	5.00 ± 0.74	1.08 ± 0.39		6.99 ± 0.51	2.93 ± 0.14		4.00 ± 0.22	5.76 ± 0.31
	FAMILY							
	CTR_T0	CTR_T1		BM_T0	BM_T1		HA_T0	HA_T1
Acetobacteraceae	0.75 ± 0.33	0.73 ± 0.04		0.40 ± 1.34	1.02 ± 0.63		0.39 ± 1.68	0.27 ± 9.46
Bartonellaceae	3.38 ± 3.29	11.08 ± 12.70		1.11 ± 0.93	1.86 ± 1.59		1.53 ± 1.51	15.08 ± 17.04
Bifidobacteraceae	10.58 ± 1.51	11.37 ± 1.39		10.16 ± 1.97	10.96 ± 2.88		11.61 ± 3.26	7.92 ± 4.27
Lactobacillaceae	55.12 ± 8.43	52.36 ± 6.83		57.90 ± 8.58	49.36 ± 10.99		54.28 ± 11.84	50.76 ± 14.76
Neisseriaceae	5.99 ± 2.09	6.06 ± 1.50		4.73 ± 1.98	10.13 ± 5.77		6.08 ± 2.36	5.29 ± 2.22
Orbaceae	19.18 ± 6.16	17.32 ± 4.82		18.71 ± 6.74	23.74 ± 6.54		22.12 ± 7.61	14.92 ± 4.33
Other_families	5.00 ± 1.55	1.08 ± 0.31		6.99 ± 5.77	2.93 ± 1.29		4.00 ± 1.45	5.76 ± 1.95
	GENUS							
	CTR_T0	CTR_T1		BM_T0	BM_T1		HA_T0	HA_T1
*Apilactobacillus*	0.00 ± 0.00	0.00 ± 0.00		0.00 ± 0.00	0.00 ± 0.00		0.00 ± 0.01	0.00 ± 0.00
*Bartonella*	3.38 ± 3.25	11.08 ± 12.70		1.11 ± 0.97	1.86 ± 1.61		1.53 ± 1.52	15.08 ± 17.04
*Bifidobacterium*	10.58 ± 1.48	11.37 ± 1.28		10.16 ± 1.91	10.96 ± 2.79		11.61 ± 3.31	7.92 ± 4.33
*Bombilactobacillus*	13.88 ± 3.87	10.64 ± 2.94		15.00 ± 1.89	7.61 ± 2.44		15.20 ± 6.36	8.39 ± 4.05
*Commensalibacter*	0.75 ± 0.64	0.73 ± 0.51		0.40 ± 0.41	1.02 ± 0.95		0.39 ± 0.70	0.27 ± 0.21
*Frischella*	5.27 ± 2.38	5.58 ± 2.11		4.97 ± 2.79	6.69 ± 3.49		6.20 ± 2.76	4.59 ± 2.70
*Gilliamella*	13.91 ± 5.25	11.74 ± 3.57		13.74 ± 4.60	17.06 ± 5.37		15.92 ± 6.79	10.33 ± 2.41
*Lactobacillus*	41.24 ± 8.76	41.72 ± 3.98		42.91 ± 8.64	40.88 ± 9.91		39.07 ± 11.67	42.37 ± 11.59
*Plantilactobacillus*	0.00 ± 0.00	0.00 ± 0.00		0.00 ± 0.00	0.87 ± 0.66		0.00 ± 0.00	0.01 ± 0.01
*Snodgrassella*	5.99 ± 2.16	6.06 ± 1.50		4.73 ± 1.97	10.13 ± 5.80		6.08 ± 2.28	5.29 ± 2.39
Other_genus	5.00 ± 1.79	1.08 ± 0.62		6.99 ± 5.35	2.93 ± 2.10		4.00 ± 1.52	5.76 ± 8.64

**Table 3:** Beta -diversity analysis based on weighted UniFrac matrix. Unweighted UniFrac is not reported for no significant comparisons. *p*-values are reported for the considered 11 comparisons both with and without Bonferroni correction (p${}_{corr})$. ** significant values (*p* < 0.05).

Group 1	Group 2	*p*	*p* ${}_{corr}$
Weighted UniFrac			
CTR_T0 vs. CTR_T0	CTR_T1 vs. CTR_T1	0.085	0.935
HA_T0 vs. HA_T0	HA_T1 vs. HA_T1	0.012	0.132
BM_T0 vs. BM_T0	BM_T0 vs. BM_T1	0.936	1
CTR_T0 vs. CTR_T1	HA_T0 vs. HA_T1	0.002	0.022 **
CTR_T0 vs. CTR_T1	BM_T0 vs. BM_T1	0.014	0.154

**Table 4:** Significant variation among microbial groups at phyla, family, genus and species level according to the presented trials and experimental conditions.

	BM	HA	CTR
Phyla	Other_phyla ↓		
Family	Neisseraceae ↑ Other_families ↓		Other_families ↓
Genus	*Snodgrassella*↑ *Bombilactobacillus* ↓	*Bartonella*↑ *Bombilactobacillus* ↓	
Species	*S. alvi*↑ *B. mellifer* ↓ *L. plantarum*↑ *B. indicum*↑ *L. jhonsoniiDan92*↑		

## Data Availability

NGS Sequencing data have been submitted to NCBI repository under the Sequence Read Archive (SRA) databases under accession numbers: SAMN16442367-SAMN16442372; SAMN16442385-SAMN16442390; SAMN16442403-SAMN16442408; SAMN16442421-SAMN16442426; SAMN16442438-SAMN16442443 and SAMN16442456-SAMN16442461 (Bioproject n° PRJNA669646). Elaborated data presented in this study are available on reasonable request from the corresponding author.

## Abbreviations

The following abbreviations are used in this manuscript:

ANOVA	Analysis of variance
BM	Bacterial mixture
CFU	Colony forming unit
CTR	Control
HA	HiveAlive^TM^
NGS	Next Generation Sequencing
OTU	Open taxonomic unit
PCA	Principal component analysis
PD	Phylogenetic diversity
qPCR	Quantitative Polymerise chain reaction
T0	Experiment begins
T1	Experiment end
